# Applications of the Stroop Paradigm in Tinnitus: a Scoping Review

**DOI:** 10.1002/brb3.71094

**Published:** 2025-11-21

**Authors:** Anna Carolina Marques Perrella de Barros, Daniela Gil, Flávia Alencar de Barros Suzuki, Andreia Cristina Feitosa do Carmo, Ektor Tsuneo Onishi, Fátima Cristina Alves Branco‐Barreiro

**Affiliations:** ^1^ Department of Speech‐Language‐Hearing Sciences Universidade Federal de São Paulo – UNIFESP, Escola Paulista De Medicina São Paulo SP Brazil; ^2^ Tinnitus Clinic – Department of Otorhinolaryngology and Head and Neck Surgery Universidade Federal de São Paulo – UNIFESP, Escola Paulista De Medicina São Paulo SP Brazil; ^3^ São Paulo Campus Library Universidade Federal de São Paulo – UNIFESP, Escola Paulista De Medicina São Paulo SP Brazil

**Keywords:** attention, cognition, Stroop test, tinnitus

## Abstract

**Introduction:**

The Stroop paradigm is important for assessing top‐down attentional control. Considering that tinnitus has been linked to cognitive dysfunctions, the present study intends to investigate Stroop's application in the tinnitus population.

**Methods:**

This is a scoping review, following PRISMA‐ScR guidelines, performed until December 2024. Searches were conducted in the MEDLINE (PubMed), Embase (Elsevier), PsycINFO (APA), SCOPUS (Elsevier), and Web of Science (Clarivate Analytics) databases. Studies were selected based on the following criteria: Types of participants: subjects with tinnitus (with no age limit); types of intervention: the Stroop paradigm applied to tinnitus population, isolated or combined with other evaluations or interventions for the tinnitus; types of study: original articles of different designs, with no language and publication date limits. Studies that used evaluations or intervention forms other than the Stroop paradigm, papers exclusively on other health conditions, congress or conferences’ material, and studies not available in the database and/or as a full text were excluded. Results were presented and qualitatively assessed based on the Critically Appraised Topics (CAT).

**Results:**

369 records were identified in the searching stage, and 22 studies were selected for data extraction. Author/date, Stroop paradigm application, Stroop methodology adopted, outcomes measures for Stroop, and level of evidence were extracted. The most common application for Stroop in tinnitus subjects was cognitive function assessment, followed by neuronal connectivity assessment during the task, development of auditory training strategies, and measurement before and after surgical treatment. The main methodology adopted for Stroop was based on conventional Stroop tasks.

**Conclusion:**

Stroop applications to the tinnitus population were diverse, considering methodologies and finalities. Results showed that Stroop can highlight top‐down cognitive control in the tinnitus population. However, the current body of evidence is limited in quality and consistency, highlighting the need for more rigorous and standardized research to better inform its clinical applicability.

## Graphical abstract

This study examines the application of the Stroop test in individuals with tinnitus, highlighting its significance in assessing cognitive function and informing audiological rehabilitation strategies. Given the growing body of evidence linking tinnitus with cognitive load and attentional deficits, our study provides valuable insights into how cognitive assessment tools can contribute to a more comprehensive understanding of tinnitus‐related cognitive challenges. Additionally, our findings highlight existing limitations in current literature and provide directions for future research.

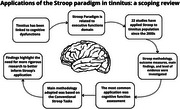

## 1 Introduction

The Stroop test was proposed by John Ridley Stroop in 1935. Test development was based on studies about effects of interference in logical associations. It assesses the power to recall associative links, through response patterns or acquired habits, in comparison with the strength of the inhibitory power (Stroop 1935). Judgment for the requested task is related to a specific dimension of the stimulus, which is not consistent with what is expected, and, therefore, it's necessary to resist against strong stimuli of associative responses that are naturally oppressive (Logan 1980).

Originally, tasks included reading words referring to color names, written in different colors and naming the colors in which the words were written, which were color names written in different colors (Stroop 1935). Time measured to read a list of words is not significantly affected if the words are written in incongruent colors, but time measured to name colors of a stimulus is reliably slower if the stimulus is the name of an incongruent color (Washburn 2016). The increase in reaction time and execution errors, caused by the presence of conflict, represent measures of the interference effect studied in the Stroop paradigm (Stroop 1935).

Stroop constitutes an ideal behavioral testing platform to assess the competition between behavioral stimulus control and cognitive control of attention. There is competition between automatic and controlled processing. Automatic behavior is rapid, stereotyped, inflexible, and effortless or with little effort in response to highly practiced circumstances. Conditions that are unfamiliar or in which automatic processing may fail require attentional effort or controlled processing and make the automatic response maladaptive (Washburn 2016).

Success in information processing would depend on the focus of attention on a certain property of a stimulus and the simultaneous suppression of irrelevant information. The ability to suppress irrelevant information simultaneously with pertinent information is an important cognitive skill in several situations, and one way to measure it is through Stroop tasks, in which one dimension of the stimulus is required, and another must be ignored (Knight and Heinrich 2017).

This is a widely used psychometric test with several adaptations cited in the literature over the years, according to specific objectives of researchers (MacLeod 1991; Waechter and Brännström 2015). The Stroop paradigm is an important tool for assessing top‐down attentional control in the presence of interference (Haupt et al. 2009). The Stroop test is related to the broad cognitive domain of executive functions, more specifically, to inhibitory control or controlled attention. It is necessary to replace the dominant or automatic response and to inhibit responses because of elements or stimuli that are distractors (Webb et al. 2018).

The Stroop test provides a wide scope for deeper investigation, ranging from the mechanisms and skills implicit in the task, the variations of the test employed over time, the brain areas activated during its execution, and the types of responses evoked, among other factors, to how the results are evaluated. Besides that, the Stroop test has been applied to different populations (MacLeod 1991).

A systematic review demonstrated that tinnitus affects approximately 14% of the adult population worldwide and has an annual incidence of 1%. Prevalence of tinnitus increases with age and can reach 24% in the elderly (Jarach et al. 2022). Some subjects will suffer from tinnitus, needing clinical support. Tinnitus can be considered a disorder and lead to functional, emotional, autonomic, and cognitive disabilities (De Ridder et al. 2021).

Tinnitus is understood as a multisensory condition that goes beyond the auditory pathway and involves cognitive and emotional components (Noreña et al. 2021). Current research, which relates tinnitus to cognitive aspects, shows a viable path to a greater understanding of the mechanisms involved in the maintenance and chronicity of the symptom, given the complexity of the activated neuronal network (De Ridder et al. 2021; De Ridder et al. 2022). Studies have shown cognitive impairment in tinnitus subjects regarding different aspects of the cognitive domain (Tegg‐Quinn et al. 2016; Wang et al. 2018; Clarke et al. 2020). Cognitive impairments due to tinnitus have been attributed to the impact of the symptom on executive control of attention (Tegg‐Quinn et al. 2016).

Attention directed to tinnitus can deplete attentional resources, and tinnitus subjects can be executing a dual task all the time (Stevens et al. 2007; Edwards et al. 2022). It was demonstrated that the dorsal anterior cingulate cortex, activated in Stroop tasks, has a more generalized processing function and can interfere with brain function at all (To et al. 2017). As the Stroop test is related to executive functions, more specifically controlled attention and inhibitory control, it can help to understand cognitive disabilities related to tinnitus.

While some studies have explored Stroop approaches applied to tinnitus subjects, there is still a need for more research to understand the efficacy and applicability of Stroop paradigms in tinnitus assessment and management. This scoping review aims to map the existing literature on the application of the Stroop paradigm in tinnitus populations, identifying methodologies, outcome measures, and gaps in current research.

### 1.1 Material and Methods

This scoping review followed the quality parameters of the Preferred Reporting Items for Systematic Reviews and Meta‐Analyses extension for Scoping Reviews (PRISMA‐ScR) (Tricco et al. 2018). Its protocol was registered in the Open Science Framework (OSF) under https://doi.org/10.17605/OSF.IO/P9GFY.

### 1.2 Eligibility Criteria

Studies were selected based on the criteria described in Table 1.

**TABLE 1:** Eligibility criteria.

Inclusion criteria	Population: adults with tinnitus (with no superior age limit); types of intervention: Stroop paradigm applied to tinnitus population, isolated or combined with other evaluations or interventions for tinnitus; comparison: Stroop's methodology applied; outcome: measures as a function of time and analysis related to the number of errors; types of study: original articles of different designs, such as descriptive, exploratory, and experimental studies; primary studies; systematic reviews; meta‐analyses; and guidelines, with no language limits. No publication date limit was set to retrieve articles.
Exclusion criteria	Studies that used evaluations or intervention forms other than the Stroop paradigm, papers exclusively on other health conditions, congress or conferences’ material, and studies that were not available in the database and/or as a full text.

### 1.3 Search

Terms from the Medical Subject Heading (MeSH) vocabulary and free terms were used (Table 2); a search was conducted in MEDLINE (PubMed), Embase (Elsevier), PsycINFO (APA), SCOPUS (Elsevier), and Web of Science (Clarivate Analytics) and updated until December 2024. Search strategies developed by the authors followed PRESS—Press Review Electronic Search Strategies recommendations (McGowan et al. 2016). The sample was selected by convenience, including all studies that met the inclusion criteria.

**TABLE 2:** Search strategy in the databases.

Database	Searching strategy
PubMed	(((("Stroop test"[MeSH Terms] OR "Stroop task"[Text Word]) AND "Stroop effect"[Text Word]) AND "Stroop paradigm"[Text Word]) OR ("neuropsychological tests"[Text Word] AND ("attention"[Text Word] OR "cognition"[Text Word]))) AND ("tinnitus"[MeSH Terms] OR "tinnitus"[All Fields])
Embase	("Stroop test"/exp OR "stroop color and word test": ti, ab, kw OR "Stroop color naming task": ti, ab, kw OR "Stroop color naming test": ti, ab, kw OR "Stroop color word conflict test": ti, ab, kw OR "Stroop color word test": ti, ab, kw OR "Stroop color and word test": ti, ab, kw OR "Stroop color naming task": ti, ab, kw OR "Stroop color naming test": ti, ab, kw OR "Stroop color word conflict test": ti, ab, kw OR "Stroop color word test": ti, ab, kw OR "Stroop effect": ti, ab, kw OR "Stroop effects": ti, ab, kw OR "Stroop interference": ti, ab, kw OR "Stroop paradigm":ti, ab, kw OR "Stroop paradigms": ti, ab, kw OR "Stroop performance": ti, ab, kw OR "Stroop task": ti, ab, kw OR "Stroop tasks": ti, ab, kw OR "Stroop test": ti, ab, kw OR "Stroop tests": ti, ab, kw OR "Stroop word color test": ti, ab, kw OR "Stroop word color test": ti, ab, kw OR "Stroop`s color word conflict test": ti, ab, kw OR "Stroop`s color word conflict test": ti, ab, kw OR "Stroop`s test": ti, ab, kw) AND "tinnitus"
SCOPUS	(("Stroop test" OR "Stroop task") AND "stroop effect" AND "Stroop paradigm") OR ("neuropsychological tests" AND ("attention" OR "cognition")) AND ("tinnitus")
Web of Science	(("Stroop test" OR "Stroop task") AND "Stroop effect" AND "Stroop paradigm") OR ("neuropsychological tests" AND ("attention" OR "cognition")) AND ("tinnitus")
PsycINFO	((TX "Stroop test" OR TX "Stroop task") AND TX "Stroop effect" AND TX "Stroop paradigm") OR (TX "neuropsychological tests" AND (TX "attention" OR TX "cognition")) AND (DE "tinnitus" OR TX "tinnitus")

### 1.4 Selection of Sources of Evidence

**FIGURE 1:**
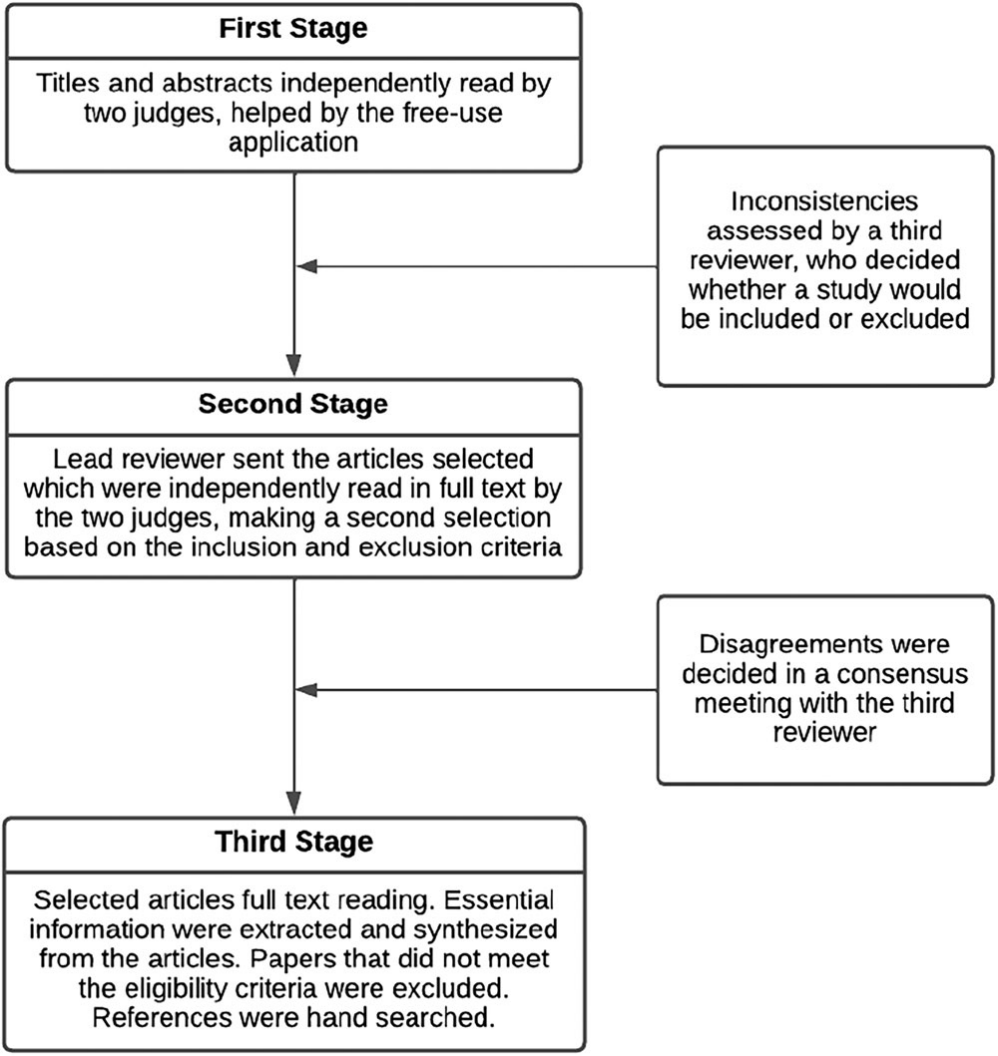
Three‐Stage process for studies’ selection.

Rayyan QCRI was used to select studies and identify duplicates in a three‐stage process, as described in Figure 1.

In the first stage, titles and abstracts were independently read by two judges (ACMPB and FABS), facilitated by the free‐use application. A third reviewer (FCABB) assessed inconsistencies between the two judges and decided whether a study would be included or excluded.

In the second stage, the lead reviewer sent the articles selected by reading the titles and abstracts, which were independently read in full text by the two judges, making a second selection based on the inclusion and exclusion criteria. In case of disagreements, the third reviewer decided in a consensus meeting whether the study would be included or excluded.

The third stage consisted of reading in full text the selected articles. The retrieved articles were imported into the EndNote reference manager, where they were stored. An online spreadsheet was developed for the reviewers to fill in with the essential information extracted from the articles and hence develop a synthesis on the topic. Papers that did not meet the eligibility criteria were excluded. Then, the references in the selected papers were hand‐searched for articles that might be included in the present study.

### 1.5 Data Extraction

Data was extracted with an instrument developed for the study and categorized according to the CASP recommendation (Barends et al. 2017). Results were described and summarized according to the objectives of this review and qualitatively assessed based on the Critically Appraised Topics (CAT), which makes critical analyses of the level of evidence of selected articles (Williams et al. 2020).

The instrument informs the study design when it is not explicitly reported by the authors and establishes the studies’ level of reliability, considering methodological adequacy (Table 3) and methodological quality (strengths and weaknesses of the study). Studies’ level of reliability was classified into very high (Level A+), high (Level A), moderate (Level B), limited (Level C), low (Level D), or very low (Level D‐).

**TABLE 3:** Classification of methodological adequacy used in the critically appraised topics.

Study design	Level
Systematic review or meta‐analysis of randomized controlled studies	AA
Systematic review or meta‐analysis of non‐randomized controlled and/or before‐after studies	A
Randomized controlled study	
Systematic review or meta‐analysis of controlled studies without a pretest or uncontrolled study with a pretest	B
Non‐randomized controlled before‐after study	
Interrupted time series	
Systematic review or meta‐analysis of cross‐sectional studies	C
Controlled study without a pretest or uncontrolled study with a pretest	
Cross‐Sectional study (survey)	D
Case studies, case reports, traditional literature reviews, theoretical papers	D‐

## 2 Results

### 2.1 Literature Search Results

**FIGURE 2:**
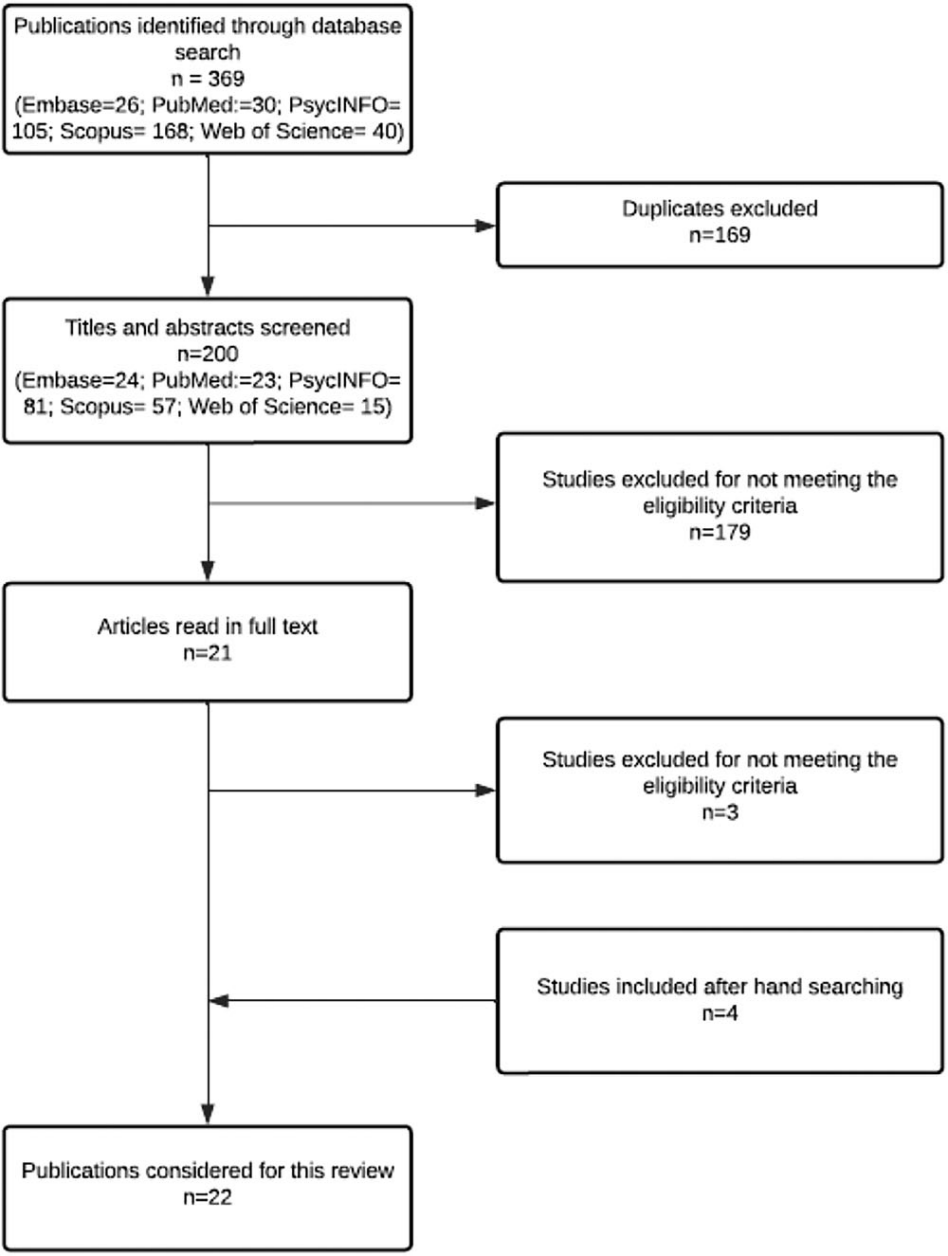
Results’ flowchart.

A total of 369 articles were identified in databases, 169 duplicates were excluded, and the remaining 200 articles, whose titles and abstracts were read. Then, 179 articles were excluded for not meeting the eligibility criteria, comprising 21 selected papers. These articles were read in full, and three of them were excluded for not meeting the eligibility criteria. The references were hand‐searched, and four additional articles were found that met the eligibility criteria, resulting in 22 articles included in this review. Figure 2 shows the selection process.

Publication time varied between 2000 and 2024. All studies found had applied the Stroop to adults or elderly subjects (Waechter and Brännström 2015; Stevens et al. 2007; Edwards et al. 2022; To et al. 2017; Andersson et al. 2005; Andersson et al. 2000; Jackson et al. 2014; Araneda et al. 2015; Araneda et al. 2018; Golm et al. 2016; James et al. 2017; Gonendik et al. 2021; Leong et al. 2020; Brueggemann et al. 2021; Emadi et al. 2023a; Emadi et al. 2023b; Emadi et al. 2022; Ghodratitoostani et al. 2022; Sommerhalder et al. 2023; Sharma et al. 2023; Sharma et al. 2022; Deniz‐Sakarya et al. 2024).

### 2.2 Stroop Applications in Tinnitus Subjects

The most widely common application of Stroop was the assessment of cognitive function (*n* = 14) (Waechter and Brännström 2015; Stevens et al. 2007; Edwards et al. 2022; Andersson et al. 2005; Andersson et al. 2000; Jackson et al. 2014; Araneda et al. 2015; Gonendik et al. 2021; Leong et al. 2020; Brueggemann et al. 2021; Sommerhalder et al. 2023; Sharma et al. 2023; Sharma et al. 2022; Deniz‐Sakarya et al. 2024), followed by the assessment of neuronal connectivity in relation to the task (*n* = 4) (Araneda et al. 2018; Golm et al. 2016; James et al. 2017; Ghodratitoostani et al. 2022), Stroop tasks usage for audiological treatment of tinnitus (*n* = 3) (Emadi et al. 2023a; Emadi et al. 2023b; Emadi et al. 2022) and Stroop Test usage as a measure before and after physician treatment (surgery) (*n* = 1) (To et al. 2017).

The application procedure was mostly based on the Stroop Conventional Test (*n* = 11) (Stevens et al. 2007; Edwards et al. 2022; Andersson et al. 2000; Jackson et al. 2014; James et al. 2017; Gonendik et al. 2021; Brueggemann et al. 2021; Sommerhalder et al. 2023; Sharma et al. 2023; Sharma et al. 2022 Deniz‐Sakarya et al. 2024), which requires word color reading and color naming tasks. Emotional Stroop was applied in five (*n* = 5) studies (To et al. 2017; Andersson et al. 2005; Andersson et al. 2000; Golm et al. 2016; Ghodratitoostani et al. 2022), and it was based on emotional valence words related to tinnitus (Andersson et al. 2005; Andersson et al. 2000; Golm et al. 2016; Ghodratitoostani et al. 2022) or words of physical or emotional threats (To et al. 2017, Andersson et al. 2000). Counting Stroop was used in three (*n* = 3) studies (Waechter and Brännström 2015; To et al. 2017; Leong et al. 2020) and usually requires symbols of numbers in a counting task that are incongruent or congruent to the number represented and visually presented; for instance, the number two is presented twice (congruent) or four times (incongruent). It was also performed with emotional words, and the participant had to count the number of presented words (To et al. 2017). Spatial Stroop was adopted in two (*n* = 2) studies (Araneda et al. 2015; Araneda et al. 2018) in which “right” and “left” words were presented visually or auditorily congruently or incongruently to the represented side for the participant perspective.

Auditory Stroop training strategies were employed in three (*n* = 3) studies (Emadi et al. 2023a; Emadi et al. 2023b; Emadi et al. 2022). Tasks were developed based on acoustic‐semantic conflicts: the words “male” or “female” auditorily presented by male and female speakers, the words “loud” or “soft” auditorily presented in soft or loud levels, and the words “long” and “short” auditorily presented in different duration times. It was performed in four (Emadi et al. 2023b) or six (Emadi et al. 2023a; Emadi et al. 2022) sessions, with a progressive increase in the level of difficulty.

Neuronal connectivity during Stroop tasks was assessed by functional magnetic resonance imaging (Araneda et al. 2018; Golm et al. 2016; James et al. 2017) and by electroencephalography (Ghodratitoostani et al. 2022) to map neuronal activity during attentional conflict (Araneda et al. 2018; James et al. 2017; Ghodratitoostani et al. 2022) and to understand intervention effects in neuronal plasticity (James et al. 2017; Ghodratitoostani et al. 2022).

Main characteristics of Stroop applications in tinnitus subjects were described in detail and categorized as follows: author/date, Stroop paradigm application, Stroop methodological approach adopted, outcome measures for Stroop, and level of evidence (according to qualitative analysis with CAT) (Table 4). Detailed information on study design, outcome measures, participants, sample characteristics, and main findings is shown in Table 5.

**TABLE 4:** Main characteristics of Stroop application in tinnitus subjects.

Reference	Applicability	Applied methodology based on	Outcome measures	Reliability
Andersson et al. (2000)	Cognitive function assessment	Conventional and Emotional Stroop	Total time	Low (60%)
Andersson et al. (2005)	Cognitive function assessment	Emotional Stroop, with and without noise presentation	Reaction time	Low (60%)
Stevens et al. (2007)	Cognitive function assessment	Conventional Stroop	Reaction time, number of errors	Low (60%)
Jackson et al. (2014)	Cognitive function assessment	Conventional Stroop	Reaction time, error rate	Low (60%)
Waechter, Brännström (2015)	Cognitive function assessment	Counting Stroop	Reaction time, accuracy	Low (60%)
Araneda et al. (2015)	Cognitive function assessment	Spatial Stroop	Reaction time, accuracy	Low (60%)
Golm et al. (2016)	Neural connectivity assessment in response to the task	Emotional Stroop	Reaction time	Low (60%)
James et al. (2017)	Neural connectivity assessment in response to the task	Conventional Stroop	N/A	High (90%)
To et al. (2017)	Before and after treatment assessment	Cognitive and Emotional Counting Stroop	Reaction time, number of errors	Limited (70%)
Araneda et al. (2018)	Neural connectivity assessment in response to the task	Spatial Stroop	Response time, Stroop effect	Low (60%)
Leong et al. (2020)	Cognitive function assessment	Counting Stroop	Reaction time	Low (60%)
Gonendik et al. (2021)	Cognitive function assessment	Conventional Stroop, with and without noise presentation	Total time	Low (60%)
Brueggemann et al. (2021)	Cognitive function assessment	Conventional Stroop	Processing time	Low (60%)
Emadi et al. (2022)	Audiological treatment	Auditory Stroop training	Reaction time	Moderate (80%)
Ghodratitoostani et al. (2022)	Neural connectivity assessment in response to the task	Emotional Stroop	Reaction time	High (90%)
Edwards et al. (2022)	Cognitive function assessment	Conventional Stroop	Reaction time, number of errors	Low (60%)
Sharma et al. (2022)	Cognitive function assessment	Conventional Stroop	Reaction time, Stroop effect	Low (60%)
Sommerhalder et al. (2023)	Cognitive function assessment	Conventional Stroop	Stroop effect	Low (60%)
Sharma et al. (2023)	Cognitive function assessment	Conventional Stroop	Reaction time, Stroop effect	Low (60%)
Emadi et al. (2023a)	Audiological treatment	Auditory Stroop training	Reaction time	Moderate (80%)
Emadi et al. (2023b)	Audiological treatment	Auditory Stroop training	Reaction time	Moderate (80%)
Deniz‐Sakarya et al. (2024)	Cognitive function assessment	Conventional Stroop	Completion time, number of errors, number of corrections	Low (60%)

Abbreviation: N/A, not applicable.

**TABLE 5:** Studies detailed information regarding study design, outcome measures, participants, sample characteristics, and main findings.

Study	Study design	Stroop task type	Outcome measures	Participants	Population characteristics	Main findings
Andersson et al. (2000)	Cross‐Sectional	Conventional/Emotional	Total time	Tinnitus group (*n* = 23); Control group (*n* = 23)	The age range was from 20 to 68 years, with no between‐group difference. Twenty participants in the tinnitus group had hearing loss (PTA worse ear = 31 dBHL at 0.5, 1, 2, and 3 kHz). Control group participants reported normal hearing and no tinnitus. All individuals with normal verbal ability and color vision. The mean duration of tinnitus was 6.3 years (SD = 4.1). Tinnitus was mostly localized in both ears (70%), with an average loudness of 47 dBHL (SD = 26), an average pitch of 5588 Hz (SD = 1899), and an MML of 36 dBSPL (SD = 18).	Tinnitus patients were significantly slower on all Stroop test conditions. Mean times for the tinnitus patients ranged from 71.1 to 111.1 s for the tinnitus patients, and from 56.1 to 88.8 s for the controls.
Andersson et al. (2005)	Cross‐Sectional	Emotional, with and without noise presentation	Reaction time	Tinnitus group (*n* = 104); Control group (*n* = 21)	Mean age was 45.4 years (SD = 15.6) for tinnitus participants and 37.8 years (SD = 15.8) for controls; 69 participants of the tinnitus group reported hearing loss. Control group participants reported normal hearing and no tinnitus. All individuals reported normal color vision. Participants presented a mean score of 24.2 (SD = 18.9) at TRQ.	Faster color naming was demonstrated for concern‐relevant words for tinnitus participants. Analyses of tinnitus group revealed that mean response times for tinnitus‐related words ranged from 1210 to 1230 msec, and for neutral words ranged from 1230 to 1250 msec. There was no effect of silent or noisy conditions on participants' performance.
Stevens et al. (2007)	Cross‐Sectional	Conventional	Reaction time, number of errors	Tinnitus group (*n* = 11); Control group (n=11)	The tinnitus and control groups were matched for age and verbal IQ. The age ranged from 18 to 65 years. Eight participants of the Tinnitus Group, and six participants of the Control Group, had hearing loss. Control Group participants did not experience tinnitus in the previous 6 months. The mean TQ score was 47.64 (SD=24.50). Participants had constant, mostly bilateral (n=9) tinnitus, for more than two years.	In the word reading stage, Tinnitus Group had an average completion time of 873.20 s, compared with 556.38 s for controls. To complete the color‐naming stage, participants with tinnitus took an average of 1558.71 s, compared with 912.09 s for controls. The Tinnitus Group made more errors in the color‐naming stage. Reaction time in Stroop tasks was longer in the Tinnitus Group.
Jackson et al. (2014)	Cross‐Sectional	Conventional	Reaction time, error rate	Tinnitus group (*n* = 33); Control group (*n* = 33)	Groups matched by age, sex, anxiety, and depression levels. Mean age of the Tinnitus Group was 48.18 (SD=17.07) and of the Control Group was 45.12 (SD=14.74). Tinnitus subjects presented a mean score of 7.06 (SD=2.38) at STSS (low/moderate tinnitus distress).	Subjects with tinnitus had statistically significantly longer reaction times in the Stroop task than controls. The error rate in the test was low, and there was no significant group effect on performance. Mean reaction time ranged from 966.45 ms to 1,118.79 for tinnitus subjects and from 838.04 to 986.91 for controls.
Waechter, Brännström (2015)	Cross‐Sectional	Counting	Reaction time, accuracy	Tinnitus group (*n* = 20); Control group (*n* = 20)	All participants had hearing level less than or equal to 20 dBHL; groups were matched for age, sex, anxiety, and depression. Furthermore, they had similar educational levels. The age ranged from 20 to 55 years. Tinnitus participants had tinnitus for at least 6 months, mostly bilateral (*n* = 17). Tonal and/or noise non fluctuating for 8 subjects and fluctuating for 6 subjects. Tonal and noise fluctuating for 6 subjects. The majority have severity of 2 (*n* = 17) at Klockhoff gradation.	No difference in performance was observed, considering reaction time and response accuracy, in the Stroop test from the groups.
Araneda et al. (2015)	Cross‐Sectional	Spatial	Reaction time, accuracy	Tinnitus group (*n* = 17); Control group (*n* = 17)	Groups were matched by age, sex, hearing, and education level. The age ranged from 20 to 67 years. Tinnitus participants’ THI score ranged from 22 to 82. The duration of tinnitus ranged from 8 to 240 months, and the tinnitus annoyance (VAS) from 0 to 10.	The mean reaction time for participants with tinnitus ranged from 51.56 to 119.56 ms for those with tinnitus, and from 43.36 to 59.45 ms for controls. The percentage of correct responses ranged from 95.24% to 97.65% for the Tinnitus Group and from 98.18% to 99.18% for the Control Group. The results showed slower processing speed for the auditory modality and lower response accuracy in subjects with tinnitus.
Golm et al. (2016)	Cross‐Sectional	Emotional	Reaction time	Tinnitus group, low distressed participants (*n* = 16); Tinnitus Group, highly distressed participants (*n* = 16); Control group (*n* = 16)	The groups were matched in age, sex, and hearing; the high‐suffering group had greater anxiety, depression, and lower vocabulary when compared to the other groups. All participants were less than 70 years old. Tinnitus participants had tinnitus for at least one year. Mean TQ score was 40 (SD = 6.69) for highly distressed participants, and 15 (SD = 6.28) for low distressed participants. Tinnitus loudness was 39.75 (SD = 20.99) for highly distressed participants, and 49.94 (SD = 20.77) for low distressed participants.	Neural connectivity assessment by fMRI. The Stroop task with tinnitus‐related words did not evoke more neural responses and did not imply worse performance in the tinnitus group and greater severity, but both tinnitus groups evaluated these words as negative as and more arousing than the neutral words. Highly distressed and low distressed tinnitus groups had different activation in the right insula and orbitofrontal activation. These areas were correlated with tinnitus distress.
James et al. (2017)	Non‐Controlled Before‐After study	Conventional	N/A	12 tinnitus participants	9 participants were male, mean age of 49 years (SD = 15), with subjective tinnitus for more than 6 months.	Neural connectivity assessment by fMRI. Neural activity related to attentional conflict in the Stroop task measured before the therapeutic intervention (rTMS) was a predictor of the change in tinnitus awareness through treatment. Poorer treatment response was predicted by baseline hyperactivation of bilateral prefrontal and parietal areas. The largest reduction in tinnitus awareness was correlated with baseline activity in the left dorsolateral prefrontal cortex. Changes in loudness and annoyance were correlated with right middle occipital activity.
To et al. (2017)	Non‐Controlled Before‐After Study	Cognitive/ Emotional Counting	Reaction time, number of errors	2 tinnitus participants	Participants underwent surgery modulating the dorsal anterior cingulate cortex. *Case 1* was male, 48‐year‐old, with bilateral tinnitus for 1‐year, moderate distress, after a whiplash injury. CT and MRI revealed a calcified meningioma of the right falx. *Case 2* was male, 64‐year‐old, with bilateral tinnitus for 39 years, TQ score of 68. MRI showed multiple white matter lesions.	There were few errors during the tests, and there was no difference between the pre‐ and post‐intervention error counts. There was an improvement in the subjects' reaction time performance under the Stroop test conditions after the surgical procedure.
Araneda et al. (2018)	Cross‐Sectional	Spatial	Response time, Stroop effect	Tinnitus group (*n* = 16); Control group (*n* =16)	Groups matched by age, sex, and hearing level. Additionally, there was no difference in education, anxiety and depression levels. Tinnitus participants’ THI score ranged from 20 to 76. The duration of tinnitus ranged from 18 to 253 months, and the tinnitus frequency was between 2000 and 8000 Hz.	Neural connectivity assessment by fMRI. The subjects with tinnitus were slower and made more errors than the controls. The differences in response time between the incongruent and congruent series were significantly greater in the participants with tinnitus than in the control group. The auditory modality of the test was more sensitive to highlighting the differences in performance between the Tinnitus Group and the Control Group. Tinnitus participants had higher functional recruitment of the dorsolateral prefrontal cortex, the cingulate gyrus, and the ventromedial prefrontal cortex during interference conditions.
Leong et al. (2020)	Cross‐Sectional	Counting	Reaction time	Tinnitus group (*n* = 18); Control group (*n* = 15)	Groups matched by age, sex, and hearing level. Tinnitus Group mean age was 49.44 years (SD = 18.02) and Control Group mean age was 51.19 years (SD = 14.66). Tinnitus participants had tinnitus for at least 6 months. Mean score was 14.39 (SD = 11.27) at TQ. Mean tinnitus loudness (VAS) was 3.69 (SD = 2.50).	Participants with tinnitus were slower to process incongruent sequences compared to participants without the symptom. Correlation analysis between TQ and reaction time indices showed a positive and significant correlation between tinnitus severity and all attentional tasks applied.
Gonendik et al. (2021)	Cross‐Sectional	Conventional, with and without noise presentation	Total time	Tinnitus group (*n* = 40); Control group (*n* = 40)	All participants had a score greater than or equal to 24 on the Mini‐Mental State Examination and a PTA (0.5, 1, 2, and 4 kHz) less than or equal to 25 dB HL. The group of participants with tinnitus presented hearing loss at the frequency at which the tinnitus pitch was measured (mean 42.87 dB HL, standard deviation 23.93). There was no difference in age and sex between the groups. Tinnitus participants had mean THI score of 41.90 (SD = 21.00), tinnitus duration of 27.60 months (SD = 9.37), tinnitus pitch of 10.610 Hz (SD=3470), and tinnitus loudness 49.37 dBHL (SD = 20.31).	Subjects with tinnitus performed worse in all stages of the test, even when there was no interference, and took longer to complete it. The presence of the acoustic stimulus favored the execution of the task for both groups.
Brueggemann et al. (2021)	Cross‐Sectional	Conventional	Processing time	107 tinnitus participants	All participants had chronic subjective tinnitus. 58 were female, and mean age was 51 (SD = 11.22). Tinnitus participants had mean TQ score of 37.73 (SD = 16) (moderate tinnitus‐related distress), tinnitus loudness of 6.55 (SD =7.22) at right ear, and of 5.70 (SD = 6.03) at left ear, tinnitus frequency of 5769.53 (SD = 2461.33) at right ear, and of 5620.43 (SD = 2539.84) at left ear.	Neural connectivity assessment by EEG. A significant correlation was observed between the degree of tinnitus‐related distress, age, hearing loss, and performance on the Stroop Interference Test. The degree of tinnitus‐related distress and age were the main predictors of cognitive performance on this task.
Emadi et al. (2022)	Non‐Randomized Controlled Study	Auditory	Reaction time	Tinnitus intervention group (*n* = 15); Tinnitus Control group (*n* = 10)	All participants had tinnitus for more than 6 months, hearing thresholds better than 35 dB HL (250‐8000 Hz). There was no difference in age, duration of tinnitus, THI scores, VAS of loudness and annoyance between groups. Tinnitus participants had tinnitus for at least 6 months. Intervention Group: Mean THI score was 48.53 (SD = 11.27), mean VAS loudness was 5.33 (SD = 0.973), and annoyance was 6.46 (SD = 1.35). Control Group: Mean THI score was 53.30 (SD = 10.34), mean VAS loudness was 5.10 (SD = 1.44), and annoyance was 6.10 (SD = 1.19).	There was a statistically significant reduction in the degree of discomfort attributed to tinnitus, measured by the VAS, and in the THI score for the group undergoing Stroop auditory training.
Ghodratitoostani et al. (2022)	Non‐Controlled Before‐After Study	Emotional	Reaction time	6 tinnitus participants	All participants had bilateral constant subjective tinnitus, normal hearing or hearing loss to a moderate degree, and normal color vision. Participants had constant bilateral subjective tinnitus.	The Emotional Stroop test was used as part of a research protocol on the dose‐response of transcranial electrical stimulation for neuromodulation in subjects with tinnitus. This is a comprehensive study, in which conventional and adaptive techniques were applied to design a Bayesian statistical model for comparison analyses.
Edwards et al. (2022)	Cross‐Sectional	Conventional	Reaction time, number of errors	Tinnitus group (*n* = 39); Control group (*n* = 39)	One participant in the Control Group and 29 participants in the Tinnitus Group self‐reported possible hearing loss. Among tinnitus participants, 27 presented a recent audiogram, which indicated the presence of mild hearing loss in the study population with tinnitus. Tinnitus participants had tinnitus for at least 3 months, and scored 17.35 (SD = 22.04) at TFI (mild tinnitus).	Participants with tinnitus had significantly longer response times than controls for both incongruent and congruent stimuli, but they also had higher levels of neuroticism. There was no difference in performance between the groups when analyzing the number of errors variable.
Sharma et al. (2022)	Cross‐Sectional	Conventional	Reaction time, Stroop effect	Tinnitus and normal hearing group (*n* = 75); Tinnitus and hearing loss group (*n* = 100)	Groups were matched by age, sex, and education level. Age ranged from 18 to 55 years. Most of the participants had mild to moderate tinnitus severity (mean THI score = 49.42, SD = 25.33). Mean tinnitus frequency ranged from 4274.62 (SD = 5025.07) to 5162.05 (4647.47). Tinnitus intensity ranged from 28.06 (SD = 28.04) to 49.04(SD = 29.78).	Tinnitus and Normal Hearing Group presented lower performance in Stroop test.
Sommerhalder et al. (2023)	Cross‐Sectional	Conventional	Stroop effect	Tinnitus Group (*n* = 25); Control Group (*n* = 25)	Groups were matched by age, sex, hearing loss, and education level. Age ranged from 23 to 58 years. Tinnitus participants had average tinnitus duration of 132.96 months (SD=93.42), average loudness of 38.21dBSPL, and average pitch at 7440 Hz (SD=3260). Mean THI score was 30.80 (SD=17.91).	Tinnitus Group had a worse performance in Stroop Test, with a large effect size. Mean Stroop effect was 126.72 ms (SD = 86.27) for the Tinnitus Group and 64.90 ms (SD = 46.09) for the Control Group.
Sharma et al. (2023)	Cross‐Sectional	Conventional	Reaction time, Stroop effect	Tinnitus and normal hearing group (n=75); Tinnitus and hearing loss group (*n* = 100)	Groups were matched by age, sex, and education level. Age ranged from 18 to 55 years. Most of the participants had mild to moderate tinnitus severity (mean THI score= 49.42, SD=25.33). Mean tinnitus frequency ranged from 4274.62 (SD=5025.07) to 5162.05 (4647.47). Tinnitus intensity ranged from 28.06 (SD=28.04) to 49.04(SD‐29.78).	Bilateral tinnitus participants had worse selective attention performance compared to unilateral tinnitus.
Emadi et al. (2023a)	Non‐Randomized controlled study	Auditory	Reaction time	Tinnitus intervention group (tDCS + Auditory Stroop Training) (*n* = 17); Tinnitus Control group (Sham tDCS + Auditory Stroop Training) (*n* = 17)	All participants had tinnitus for more than 6 months, hearing thresholds better than 35 dB HL (250‐8000 Hz). Before the intervention, the groups presented scores without statistically significant differences in the THI, VAS of tinnitus intensity and annoyance. Tinnitus participants had tinnitus for at least 6 months. Intervention Group: Mean THI score was 50.88. Control Group: Mean THI score was 49.42.	Both groups showed significant reductions in THI scores and VAS scores for tinnitus intensity and annoyance after the interventions. tDCS and auditory stroop training had a greater effect on reducing THI scores. There was a significant correlation between the reduction in reaction time in the incongruent series of auditory Stroop conditions, the decrease in THI scores, and the improvement in tinnitus‐related annoyance.
Emadi et al. (2023b)	Non‐Randomized Controlled Study	Auditory	Reaction time	Tinnitus intervention group (*n* = 15); Tinnitus Control group (*n* = 15)	All participants had tinnitus for more than 6 months. Groups were matched by age, sex, and hearing levels. Before the intervention, the groups presented scores without statistically significant differences in the THI and mean duration of tinnitus. Tinnitus participants had tinnitus for at least 6 months. Before training (Intervention Group): Mean THI score was 51.78 (SD=12.23), mean VAS loudness was 5.71, and annoyance was 7.73.	There was an improvement in quality of life, THI scores, VAS of annoyance, and reaction time on the Stroop task after the Auditory Stroop Training in the Tinnitus Intervention Group. There was a positive correlation between reaction time on the Stroop task, improvement in quality of life, THI score, and VAS of annoyance.
Deniz‐Sakarya et al. (2024)	Cross‐Sectional	Conventional	Completion time, number of errors, number of corrections	Tinnitus group (*n* = 30); Control group (*n* = 30)	All participants were 18 to 55 years, pure tone average less than or equal 25 dB HL, and normal cognitive abilities (Montreal Cognitive Assessment Test). There was no difference between groups in age, sex, educational level, and depressive symptom score. Tinnitus participants had tinnitus for at least 6 months. THI mean score was 40.73 (SD = 26.93), tinnitus loudness ranged from 7 to 50 dBHL, tinnitus pitch ranged from 250 to 18000 Hz, and MML ranged from 10 to 55 dBHL. Mean loudness VAS was 4.7 (SD = 2.00). Mean VAS annoyance was 5.20 (SD = 2.33). 57% (*n* = 17) of the participants had bilateral tinnitus.	Both groups showed similar results regarding completion time, number of errors and corrections in the Stroop Test.

Abbreviations: CT, computerized tomography; dBHL, decibel hearing level; dBSPL, decibel sound pressure level; EEG, electroencephalography; (f)MRI, (functional) magnetic resonance imaging; Hz, Hertz; IQ, intelligence quotient; MML, minimum masking level; ms, milliseconds; N/A, not applicable; PTA, Pure Tone Average; rTMS, Repetitive Transcranial Magnetic Stimulation; s, seconds; SD, standard deviation; STSS, Subjective Tinnitus Severity Scale; tDCS, Transcranial Direct Current Stimulation; TFI, Tinnitus Functional Index; THI, Tinnitus Handicap Inventory; TQ, Tinnitus Questionnaire; TRQ, Tinnitus Reactions Questionnaire; VAS, Visual Analog Scale.

### 2.3 CAT Reliability Analysis

Reliability analysis by the CAT instrument revealed the level of evidence ranging from low (60%) to high (90%). Cross‐sectional studies (Waechter and Brännström 2015; Stevens et al. 2007; To et al. 2017; Andersson et al. 2005; Andersson et al. 2000; Jackson et al. 2014; Araneda et al. 2015; Araneda et al. 2018; Golm et al. 2016; Gonendik et al. 2021; Leong et al. 2020; Brueggemann et al. 2021; Sommerhalder et al. 2023; Sharma et al. 2023; Sharma et al. 2022; Deniz‐Sakarya et al. 2024) were classified as “Level D” considering CAT analyses, which means that the trustworthiness and reliability of the study were considered low (60%). A non‐controlled before‐after study (To et al. 2017) was considered “Level C” and had limited trustworthiness (70%). Non‐randomized controlled studies (Emadi et al. 2023a; Emadi et al. 2023b; Emadi et al. 2022) were moderately appropriate (Level B) and had 80% trustworthiness. Randomized controlled studies (James et al. 2017; Ghodratitoostani et al. 2022) were considered appropriate and had higher trustworthiness (90%).

### 2.4 Studies Participants

The number of participants in studies varied between 2^19^ and 175^40^. Studies that applied the Stroop Test for cognitive function assessment had the most variability regarding sample size, ranging from 22^17^ to 175^40^ subjects. Main characteristics of these studies are shown in Table 6. Studies that investigate neuronal connectivity during Stroop tasks had sample sizes ranging from 6^37^ to 48^29^. Intervention studies had sample sizes between 25^36^ and 34^34^. Two participants were included in the study that applied Stroop measures before and after surgical intervention (To et al. 2017). All participants were adults and/or elderly subjects. A limited number of studies comprising cognitive function assessment had control groups with controlled variables such as age, sex, anxiety, depression, and educational level (Waechter and Brännström 2015; Araneda et al. 2015; James et al. 2017; Leong et al. 2020) (Tables 5 and 6).

**TABLE 6:** Main characteristics of studies that applied Stroop to cognitive assessment of tinnitus subjects.

Cognitive function assessment	
Participants	Adults (Waechter and Brännström 2015; Stevens et al. 2007; Jackson et al. 2014; Araneda et al. 2018; Golm et al. 2016; Gonendik et al. 2021; Sommerhalder et al. 2023; Sharma et al. 2023; Sharma et al. 2022); Adults and Elderly Subjects^,24, 25, 27, 32, 33^
Sample size	22 participants (Stevens et al. 2007); 32 participants (Araneda et al. 2018); 33 participants (Leong et al. 2020); 34 participants (Araneda et al. 2015); 40 participants (Waechter and Brännström 2015); 46 participants (Andersson et al. 2000); 48 participants (Golm et al. 2016); 50 participants (Sommerhalder et al. 2023); 60 participants (Deniz‐Sakarya et al. 2024); 66 participants (Jackson et al. 2014); 78 participants (Edwards et al. 2022); 80 participants (Gonendik et al. 2021); 107 participants (Brueggemann et al. 2021); 125 participants (Andersson et al. 2005); 150 participants (Sharma et al. 2023); 175 participants (Sharma et al. 2022)
Stroop's stimuli applied	Visual (Waechter and Brännström 2015; Stevens et al. 2007; Edwards et al. 2022; Andersson et al. 2005; Andersson et al. 2000; Gonendik et al. 2021; Leong et al. 2020; Brueggemann et al. 2021; Sommerhalder et al. 2023; Sharma et al. 2023; Sharma et al. 2022; Deniz‐Sakarya et al. 2024); Verbal (Stevens et al. 2007; Edwards et al. 2022; Andersson et al. 2005; Andersson et al. 2000; Jackson et al. 2014; Araneda et al. 2015; Araneda et al. 2018; Golm et al. 2016; Gonendik et al. 2021; Leong et al. 2020; Brueggemann et al. 2021; Sommerhalder et al. 2023; Sharma et al. 2023; Sharma et al. 2022; Deniz‐Sakarya et al. 2024); Auditory (Araneda et al. 2015; Araneda et al. 2018); Non‐Verbal (Waechter and Brännström 2015)
Stroop test's administration	Alone (Waechter and Brännström 2015; Edwards et al. 2022; Andersson et al. 2005; Andersson et al. 2000; Araneda et al. 2015; Gonendik et al. 2021); associated with other cognitive tasks (Stevens et al. 2007; Jackson et al. 2014; Leong et al. 2020; Brueggemann et al. 2021; Sommerhalder et al. 2023; Sharma et al. 2023; Sharma et al. 2022; Deniz‐Sakarya et al. 2024)
Control Group pairment or controlled variables	Age and sex (Andersson et al. 2000; Gonendik et al. 2021); age and verbal IQ (Stevens et al. 2007); age, sex, and hearing level (Golm et al. 2016; Leong et al. 2020); age, sex, and anxiety and depression level (Jackson et al. 2014); age, sex, education, and hearing level (Sommerhalder et al. 2023; Deniz‐Sakarya et al. 2024); age, sex, anxiety, and depression and neuroticism level (Edwards et al. 2022); age, sex, hearing, anxiety, and depression and educational levels (Waechter and Brännström 2015; Araneda et al. 2015, Araneda et al. 2018)
Main results (Tinnitus Group × Control Group)	Longer response time, total execution time, reaction time or Stroop Effect for tinnitus subjects (Stevens et al. 2007; Andersson et al. 2000; Jackson et al. 2014; Araneda et al. 2015; Araneda et al. 2018; Gonendik et al. 2021; Leong et al. 2020; Sommerhalder et al. 2023); similar number of errors, error rate or accuracy (Waechter and Brännström 2015, Edwards et al. 2022; Jackson et al. 2014; Deniz‐Sakarya et al. 2024); similar time or reaction time (Waechter and Brännström 2015; Edwards et al. 2022; Golm et al. 2016; Deniz‐Sakarya et al. 2024); more number of errors, higher error rate or less accuracy for tinnitus subjects (Stevens et al. 2007; Araneda et al. 2015); Tinnitus words were color named faster than neutral words (Andersson et al. 2005)

### 2.5 Outcome Measures

The main outcome measure adopted in studies included analysis in terms of the execution time of the Stroop task or the reaction time to the presented Stroop series (Waechter and Brännström 2015; Stevens et al. 2007; Edwards et al. 2022; To et al. 2017; Andersson et al. 2005; Andersson et al. 2000; Jackson et al. 2014; Araneda et al. 2015; Araneda et al. 2018; Golm et al. 2016; Gonendik et al. 2021; Leong et al. 2020; Brueggemann et al. 2021; Emadi et al. 2023a; Emadi et al. 2023b; Emadi et al. 2022; Ghodratitoostani et al. 2022; Sharma et al. 2023; Sharma et al. 2022; Deniz‐Sakarya et al. 2024). Analysis regarding errors performed or accuracy (percentage of correct answers) was performed in seven studies (Waechter and Brännström 2015; Stevens et al. 2007; Edwards et al. 2022; Jackson et al. 2014; Araneda et al. 2015; Araneda et al. 2018; Deniz‐Sakarya et al. 2024). Three studies used the Stroop Effect measure as an outcome mensuration (Araneda et al. 2018; Sharma et al. 2023; Sharma et al. 2022), comparing congruent and incongruent trials. One study applied an additional outcome measure that was the number of corrections during the Stroop test (Deniz‐Sakarya et al. 2024).

### 2.6 Main Findings of the Application of Stroop in Tinnitus Subjects

Most applications of the Stroop paradigm for cognitive assessment purposes have shown differences in performance in individuals with tinnitus when compared to individuals without the symptom. Tinnitus subjects had poorer performance, taking more time to perform or react to the task (Stevens et al. 2007; Edwards et al. 2022; Andersson et al. 2000; Jackson et al. 2014, Araneda et al. 2015; Araneda et al. 2018; Gonendik et al. 2021; Leong et al. 2020; Sommerhalder et al. 2023) and performing more errors (Jackson et al. 2014, Araneda et al. 2015, Araneda et al. 2018).

Some studies used words with high negative valence associated with tinnitus to enhance the sensitivity of the Stroop test (Andersson et al. 2005; Andersson et al. 2000; Golm et al. 2016; Ghodratitoostani et al. 2022). In this context, the so‐called emotional Stroop presented a facilitation effect. Subjects with tinnitus had shorter reaction times for these words than for neutral words (Table 5) (Andersson et al. 2005).

Other studies have explored the effect of using the auditory Stroop modality and comparing it to the visual modality (Araneda et al. 2015, Araneda et al. 2018), showing that the auditory modality can be more sensitive for assessing this population (Table 5). Regarding test conditions, the presence of noise during the test did not interfere with the results of one study (Andersson et al. 2005) and showed an improvement in performance for all participants in another, regardless of the presence of tinnitus (Gonendik et al. 2021), thus proving to be an irrelevant factor in the effect on performance for subjects with tinnitus.

Neural connectivity assessment in response to the Stroop task revealed that (a) neural activity related to attentional conflict in the Stroop task measured before therapeutic intervention was a predictor of change in tinnitus awareness by repetitive transcranial magnetic stimulation treatment (James et al. 2017). (b) changes in brain activity related to the Stroop effect in the dorsomedial prefrontal cortex, ventromedial prefrontal cortex, and cingulate gyrus in tinnitus subjects compared to control subjects (Araneda et al. 2018), (c) correlation between efficiency in top‐down cognitive executive control and the level of brain activity in the right dorsomedial prefrontal cortex and ventromedial prefrontal cortex in participants with tinnitus (Araneda et al. 2018), and that (d) tinnitus distress can be correlated to activation of the right insula and the orbitofrontal cortex (Golm et al. 2016).

Reaction time improvement was observed after surgical intervention in tinnitus subjects with dorsal anterior cingulate cortex abnormalities (To et al. 2017).

Some studies have explored the auditory modality of the Stroop to establish strategies for auditory training of subjects with tinnitus, either in isolation (Emadi et al. 2023b; Emadi et al. 2022) or combined with transcranial direct current stimulation (Emadi et al. 2023a). There was reaction time improvement after auditory Stroop training sessions for tinnitus subjects (Emadi et al. 2023b; Emadi et al. 2022), and reduction of the reaction time was related to a decrease in Tinnitus Handicap Inventory (THI) score and annoyance of tinnitus after training (Emadi et al. 2023a) (Table 5).

## 3 Discussion

The conscious and attentive perception of tinnitus would increase the cognitive and emotional value associated with the symptom. The cognitive‐attentional value would govern the focus of attention and be responsible for the disturbance implicit in the suffering caused by tinnitus (Ghodratitoostani et al. 2016). Tinnitus has been associated with deficient cognitive coping strategies, leading to cognitive reactions (De Ridder et al. 2022) and to impaired executive function (De Ridder et al. 2022; Clarke et al. 2020; Araneda et al. 2015; Araneda et al. 2018).

As a behaviorally relevant signal, tinnitus presents enhanced processing at the central nervous system level compared to other competitive stimuli. Considering tinnitus’ association with emotional valence, the symptom becomes the focus of selective attention (Ghodratitoostani et al. 2016).

The Stroop test is a well‐established and widely used tool for assessing attentional and inhibitory control (Webb et al. 2018; Leong et al. 2020; Brueggemann et al. 2021; Martins et al. 2023; Heinrich and Knight 2020). Initial published studies applying the Stroop paradigm to tinnitus subjects dated from 2000, accompaning the growth of knowledge regarding cognitive components related to tinnitus. In our review, we found 22 studies that administered the Stroop test or Stroop approaches to tinnitus participants, almost one paper per year, which reinforces that it must be an interesting perspective to be explored in future research.

Building upon the previously mentioned information, even in a specific population, Stroop applications can have different purposes. In the present study, we defined the main application of the Stroop paradigm in tinnitus subjects in the included studies.

The present study revealed that Stroop's most common application was cognitive function assessment (Waechter and Brännström 2015; Stevens et al. 2007; Edwards et al. 2022; Andersson et al. 2005; Andersson et al. 2000; Jackson et al. 2014; Araneda et al. 2015; Gonendik et al. 2021; Leong et al. 2020; Brueggemann et al. 2021; Sommerhalder et al. 2023; Sharma et al. 2023; Sharma et al. 2022; Deniz‐Sakarya et al. 2024) (Table 4). Among these studies, most of the methodology applied was based on the Conventional Stroop (Stevens et al. 2007; Edwards et al. 2022; Andersson et al. 2000; Jackson et al. 2014; Gonendik et al. 2021; Brueggemann et al. 2021; Sommerhalder et al. 2023; Sharma et al. 2023; Sharma et al. 2022; Deniz‐Sakarya et al. 2024). The Stroop test to evaluate cognitive function was administered alone (Waechter and Brännström 2015; Edwards et al. 2022; Andersson et al. 2005; Andersson et al. 2000; Araneda et al. 2015; Gonendik et al. 2021) or associated with different cognitive tasks to assess other cognitive skills (Stevens et al. 2007; Jackson et al. 2014; Leong et al. 2020; Brueggemann et al. 2021; Sommerhalder et al. 2023; Sharma et al. 2023; Sharma et al. 2022; Deniz‐Sakarya et al. 2024).

Noise during test execution would improve performance due to tinnitus masking. Control subjects also had a facilitation effect with noise presentation during Stroop, indicating that the effect was not specific to the tinnitus subjects (Gonendik et al. 2021). The emotional Stroop administered to tinnitus subjects had similar results to assess cognitive function independently of the word's nature, related to tinnitus annoying aspects or physical threats (Andersson et al. 2000), and independently of the noise background presence to mask tinnitus (Andersson et al. 2005).

The Counting Stroop was not able to identify performance differences when normal‐hearing tinnitus subjects were compared to control subjects (Waechter and Brännström 2015). Spatial Stroop was adapted to evaluate inhibitory control in tinnitus subjects, comparing the efficacy of two different modalities of the delivered stimuli: visual and auditory. The tinnitus and control groups had the same hearing level (Table 5). Results showed lower processing speed and lower response accuracy for the auditory modality in subjects with tinnitus, suggesting altered top‐down cognitive control (Araneda et al. 2015).

The Stroop applications for cognitive assessment were mostly based on conventional Stroop derivations. Besides that, other Stroop methodological approaches were employed to evaluate cognitive function. Our findings underscore an attempt to sensitize the Stroop test to tinnitus population assessment through tinnitus‐annoying words, auditory modality, or concomitant noise presentation. In other words, it highlights the effort to identify the most effective approach to adapt Stroop for this population and for this purpose.

During Stroop tasks, neuronal connectivity assessment showed that tinnitus subjects may require more comprehensive neuronal activation to perform the task due to executive function impairment (Araneda et al. 2018) and that neuronal activation during Stroop can be correlated with the tinnitus‐related distress network (Golm et al. 2016).

Most of the studies included adopted functional magnetic resonance imaging (fMRI) to investigate neuronal function related to Stroop (Araneda et al. 2018; Golm et al. 2016; James et al. 2017). In contrast, one study used electroencephalography (EEG) for this purpose (Ghodratitoostani et al. 2022). Cortical activation in some areas can predict tinnitus‐specific related outcomes (James et al. 2017). Furthermore, tinnitus subjects had different functional recruitment of cortical areas during Stroop performance (Araneda et al. 2018). Additionally, it was demonstrated that low‐ and highly distressed tinnitus subjects can present different activations of cortical areas implicated in emotional and salience processing (Golm et al. 2016) (Table 5). Functional neuroimaging may provide significant insights about neuronal correlates of tinnitus, although the resolution of current modalities is a challenge for conclusive directions (Isler et al. 2023).

None of the studies included have applied long‐latency auditory evoked potentials for neuronal activity assessment. The P300 can be used as a measure of cognitive processing, is linked to attentional processes, and may be considered a potential biomarker for subjective tinnitus (Cardon et al. 2020). The Stroop test is related to inhibitory control (Webb et al. 2018), which can be assessed by cortical auditory evoked potentials (Morse and Morse 2025).

Auditory Stroop training approaches showed improvement of tinnitus annoyance (Emadi et al. 2023a; Emadi et al. 2022) and quality of life (Emadi et al. 2023b), applied in isolation (Emadi et al. 2023b; Emadi et al. 2022) or combined with other intervention strategies (Emadi et al. 2023a). Auditory Stroop training combined with transcranial direct current stimulation showed an increased effect on THI scores (Emadi et al. 2023a) (Table 5). These findings show how relevant attentional control could be for tinnitus subjects’ audiological treatment, considering that this strategy applied alone already had improvements on some outcome measures. Additionally, point to a new perspective in auditory training for tinnitus already mentioned in a previous study, considering that recent studies on the topic addressed attentional factors and multisensory paths in training program activities (Barros et al. 2024). A clinical practice guideline about tinnitus diagnosis and management pointed out that distracting attention strategies adopted in hearing rehabilitation programs could benefit tinnitus habituation (Mazurek et al. 2022).

Although Stroop auditory training interventional studies included subjects with hearing loss, there was no information about the use of hearing devices during the training program. Hearing aids can influence auditory training and cognitive performance (Van Wilderode et al. 2023; Alcântara et al. 2022).

There was a consensus about time measures during Stroop tasks applied to the tinnitus population, considering that the main outcome measures applied to analyze Stroop findings were based on measurements taken over time (Waechter and Brännström 2015; Stevens et al. 2007; Edwards et al. 2022; To et al. 2017; Andersson et al. 2005; Andersson et al. 2000; Jackson et al. 2014; Araneda et al. 2015; Araneda et al. 2018; Golm et al. 2016; Gonendik et al. 2021; Leong et al. 2020; Brueggemann et al. 2021; Emadi et al. 2023a; Emadi et al. 2023b; Emadi et al. 2022; Sommerhalder et al. 2023; Sharma et al. 2023; Sharma et al. 2022; Deniz‐Sakarya et al. 2024). Analysis in terms of error occurrence was the second most used measure (Waechter and Brännström 2015; Stevens et al. 2007; Edwards et al. 2022; To et al. 2017; Jackson et al. 2014; Araneda et al. 2015; Deniz‐Sakarya et al. 2024).

Reliability was assessed by CAT, and the level of evidence of selected studies was mainly defined by study design. Most of the studies to assess cognitive function or to assess neural connectivity during Stroop tasks were cross‐sectional studies (level of evidence D, as shown in Table 3). These findings reflect the overall low methodological rigor of the included studies, as most were classified within lower levels of evidence (Table 4). As a result, the strength and generalizability of the conclusions drawn from the current literature are limited.

Intervention studies had a higher level of evidence (Table 4), but analysis revealed a need for randomized controlled trials regarding this topic. The studies that had 90% reliability employed Stroop approaches for neural connectivity assessment during a conflict attentional task purpose as an outcome measure for an intervention that was the focus of the study (James et al. 2017; Ghodratitoostani et al. 2022). In other words, the applicability of the Stroop paradigm was not the main object of these studies.

Included studies showed comprehensive possibilities to explore Stroop paradigm‐based approaches for the tinnitus population. Furthermore, there was a growing interest of researchers in Stroop applicability for tinnitus over time and a clear attempt to highlight the importance of Stroop approaches applied to the tinnitus population.

Regarding the Stroop approach adopted, most studies applied methodologies that included visual modality stimuli to the Stroop tasks. Studies that applied auditory stimulation in Stroop tasks for tinnitus subjects had employed verbal stimuli. It should be emphasized that verbal stimuli applied in these studies required more sophisticated processing, including notions of laterality, processing of verbal stimuli, stimulus recognition, and analysis of meaning. Stated differently, it could have increased the complexity of establishing the automatic association and the conflict necessary for the Stroop task. Nevertheless, it remains unclear which methodology would be the best to apply for cognitive assessment, neuronal activity assessment, audiological treatment of tinnitus, and before‐and‐after intervention assessment.

A systematic review aimed to verify the impact of tinnitus on cognitive function and found a lack of control group matching for hearing loss, anxiety, and depression in existing studies (Waechter and Brännström 2015). Some included studies presented heterogeneous clinical conditions for the Tinnitus Group and the Control Group (Table 5), which may potentially influence the interpretation and generalizability of findings.

Hearing impairment by itself is found to play an important role in cognitive function disability (Grenier et al. 2024; de Araújo et al. 2025). The association between hearing loss and tinnitus may have a significant impact on cognitive performance (Waechter and Brännström 2015; de Araújo et al. 2025; Waechter et al. 2021; Waechter et al. 2022). Additionally, some of the included studies enrolled elderly subjects, which can be a confounding factor, considering that aging is associated with an increased occurrence of hearing loss and cognitive decline.

Anxiety and depression levels may have an important effect on modulating emotional reaction to tinnitus, and studies that have not screened these conditions may mistakenly attribute cognitive difficulties to tinnitus (Tegg‐Quinn et al. 2016). Emotional characterization of tinnitus subjects is essential to address cognitive issues related to tinnitus, considering that emotional factors may mediate cognitive function in the tinnitus population (Fetoni et al. 2021; Inguscio et al. 2024).

Some studies had controlled the variables of age, sex, hearing level, educational or intellectual level, anxiety, and depression level of the sample (Waechter and Brännström 2015; Araneda et al. 2015; Araneda et al. 2018; Leong et al. 2020) (Tables 5 and 6). Subjects with tinnitus were slower (Araneda et al. 2015; Araneda et al. 2018; Leong et al. 2020) and made more errors (Araneda et al. 2015; Araneda et al. 2018) than controls when submitted to Stroop tasks, demonstrating that tinnitus itself can be a factor that interferes with the executive control of attention. Only one study found no differences in the performance of subjects with tinnitus compared to controls. The authors noted a possible influence of the applied methodology or the potential role of hearing level in the cognitive performance of subjects with tinnitus (Waechter and Brännström 2015).

The Stroop test is related to inhibitory control or controlled attention (Webb et al. 2018), but it is important to mention that other cognitive processes have been linked to tinnitus‐related disability. A systematic review found an association between tinnitus and worse cognitive performance in areas such as executive function, processing speed, short‐term memory, general learning, and retrieval (Clarke et al. 2020).

This scoping review highlights the evolving understanding of tinnitus, shifting from peripheral auditory processes to central mechanisms involving cognitive and emotional networks. The application of the Stroop test in tinnitus research has provided valuable insights into the cognitive impairments and attentional control issues faced by individuals with tinnitus. Despite methodological variability and the need for larger, more rigorous studies, the consistent use of the Stroop paradigm underscores its potential as a tool for cognitive assessment and intervention in tinnitus management.

The current body of evidence presents several limitations. We found heterogeneous data regarding tinnitus characterization among included studies. Studies demonstrated considerable methodological heterogeneity and variability in sample sizes, which affect the comparability of results. Many included studies had small samples, limiting statistical power and the generalizability of findings. These factors increase the risk of type II errors, thereby reducing confidence in conclusions and limiting the interpretation of the clinical applicability of the Stroop paradigm in the tinnitus population.

Most of the studies included in the review were cross‐sectional, which provides a snapshot of data at a single point in time. Additionally, uncontrolled variables may have influenced the results in cross‐sectional studies, potentially introducing bias. Another source of bias is that studies focused exclusively on adult or elderly participants. There may be potential biases in study selection and publication bias. Studies with positive findings are more likely to be published, which can skew the overall results. Furthermore, the selection criteria for study inclusion in the review might have introduced selection bias. This review did not include a formal risk of bias assessment, which could have enhanced the trustworthiness and validity of the findings. In addition, the search strategy did not encompass gray literature sources, potentially excluding studies with null or negative results and contributing to publication bias.

The quality of the included studies varied, with many classified as a low level of evidence (level D). This highlights the need for future research employing more rigorous and standardized methodologies. The outcome measures used to assess the Stroop test results varied across studies, which makes it difficult to compare results directly. There is a lack of standardization in the administration of the Stroop test among studies involving tinnitus patients. Consequently, the conclusions drawn in this review should be interpreted with caution. Additionally, as stated before, there is a clear need for high‐quality studies to better inform the clinical applicability of the Stroop paradigm in this population.

Future research should focus on refining these methodologies, exploring auditory Stroop applications, and conducting randomized controlled trials to establish robust evidence for clinical practice. The growing interest in this area suggests a promising avenue for improving the quality of life for those affected by tinnitus through targeted cognitive and attentional training strategies.

## 4 Conclusions

This scoping review highlights the diverse methodologies and purposes of Stroop applications in tinnitus research. The most common application was to assess cognitive function using conventional Stroop tasks. Results indicate that tinnitus subjects often perform poorer on Stroop tasks compared to non‐tinnitus subjects and exhibit different patterns of neuronal activation. Additionally, Stroop's auditory training strategies show promise in rehabilitation programs. Despite these findings, the included studies showed methodological variability and small sample sizes, which limit the generalizability of the results. Future research should focus on validating Stroop‐based protocols in controlled clinical settings and exploring their applicability to pediatric and diverse tinnitus populations. Comparing these findings with other studies reinforces the potential of Stroop applications in understanding and managing tinnitus‐related cognitive impairments.

## Author Contributions

**A.C.M.P.B**.: Conceptualization, methodology, data curation, and writing – original draft. **D.G**.: Validation, visualization, writing – review and editing. **F.A.B.S**.: Validation, visualization, writing – review and editing. **A.C.F.C**.: Methodology, investigation (search strategy design and execution), and resources. **E.T.O**.: Supervision, project administration, writing – review and editing. **F.C.A.B.B**.: Supervision, project administration, writing – review and editing. All authors approved the final manuscript.

## Funding

This research was supported by the Coordenação de Aperfeiçoamento de Pessoal de Nível Superior (CAPES).

## Ethics Statement

This scoping review used data from publicly available sources and did not involve human participants or identifiable personal data; therefore, ethical approval was not required.

## Conflicts of Interest

The authors declare no conflicts of interest.

## Data Availability

This scoping review synthesized information from previously published research articles. All data supporting the results of this review are available within the cited literature.

## References

[brb371094-bib-0001] Alcântara, Y. B. , W. W. F. Toledo , K. R. de Lima , A. T. L. Carnaúba , E. F. B. Chagas , and A. C. F. Frizzo . 2022. “Changes in Cortical Auditory Evoked Potentials in Response to Auditory Training in Elderly Hearing Aid Users: A Pilot Study.” PLOS Global Public Health 2, no. 5: e0000356. 10.1371/journal.pgph.0000356. 36962204 PMC10021855

[brb371094-bib-0002] Andersson, G. , R. Bakhsh , L. Johansson , V. Kaldo , and P. Carlbring . 2005. “Stroop Facilitation in Tinnitus Patients: An Experiment Conducted via the World Wide Web.” CyberPsychology & Behavior 8, no. 1: 32–38. 15738691 10.1089/cpb.2005.8.32

[brb371094-bib-0003] Andersson, G. , J. Eriksson , L. G. Lundh , and L. Lyttkens . 2000. “Tinnitus and Cognitive Interference.” Journal of Speech, Language, and Hearing Research 43, no. 5: 1168–1173. 10.1044/jslhr.4305.1168 11063238

[brb371094-bib-0004] Araneda, R. , A. G. De Volder , N. Deggouj , and L. Renier . 2015. “Altered Inhibitory Control and Increased Sensitivity to Cross‐modal Interference in Tinnitus During Auditory and Visual Tasks.” PLoS ONE 10, no. 3: e0120387. 25763867 10.1371/journal.pone.0120387 PMC4357462

[brb371094-bib-0005] Araneda, R. , L. Renier , L. Dricot , et al. 2018. “A Key Role of the Prefrontal Cortex in the Maintenance of Chronic Tinnitus: An fMRI Study Using a Stroop Task.” NeuroImage: Clinical 17: 325–334. 29159044 10.1016/j.nicl.2017.10.029 PMC5675730

[brb371094-bib-0006] Barends E. , Rousseau D. M. , and Briner R. B. eds. 2017. CEBMa Guideline for Critically Appraised Topics in Management and Organizations (Version 1.1). Center for Evidence‐Based Management.

[brb371094-bib-0007] Barros, A. C. M. P. , R. V. Lopes , D. Gil , A. C. F. Carmo , E. T. Onishi , and F. C. A. Branco‐Barreiro . 2024. “Auditory Training for Tinnitus Treatment: a Scoping Review.” Brazilian Journal of Otorhinolaryngology 90, no. 1: 101361. 38006725 10.1016/j.bjorl.2023.101361 PMC10709205

[brb371094-bib-0008] Brueggemann, P. , P. K. A. Neff , M. Meyer , et al. 2021. “On the Relationship Between Tinnitus Distress, Cognitive Performance, and Aging.” In Proceedings of the 2021 Prog Brain Res. (pp. 263–285). 10.1016/bs.pbr.2021.01.028 33931184

[brb371094-bib-0009] Cardon, E. , I. Joossen , H. Vermeersch , et al. 2020. “Systematic Review and Meta‐Analysis of Late Auditory Evoked Potentials as a Candidate Biomarker in the Assessment of Tinnitus.” PLoS ONE 15, no. 12: e0243785. 33332414 10.1371/journal.pone.0243785 PMC7746183

[brb371094-bib-0010] Clarke, N. A. , H. Henshaw , M. A. Akeroyd , B. Adams , and D. J. Hoare . 2020. “Associations Between Subjective Tinnitus and Cognitive Performance: Systematic Review and Meta‐analyses.” Trends in Hearing 24: 233121652091841. 10.1177/2331216520918416 PMC7243410 32436477

[brb371094-bib-0011] de Araújo, P. I. M. P. , P. L. E. S. Duarte , H. V. L. Ramos , et al. 2025. “Impact of Hearing Impairment on Cognitive Performance.” Brazilian Journal of Otorhinolaryngology 91, no. 2: 101521. 39504598 10.1016/j.bjorl.2024.101521 PMC11570816

[brb371094-bib-0012] De Ridder, D. , D. Adhia , and B. Langguth . 2021. “Tinnitus and Brain Stimulation.” In Proceedings of the 2021 International Tinnitus Seminar 249–293. 10.1007/7854_2021_219 33826134

[brb371094-bib-0013] De Ridder, D. , W. Schlee , S. Vanneste , et al. 2021. “Tinnitus and Tinnitus Disorder: Theoretical and Operational Definitions (an international multidisciplinary proposal).” Progress in Brain Research 260: 1–25. 10.1016/bs.pbr.2020.12.002. 33637213

[brb371094-bib-0014] De Ridder, D. , S. Vanneste , J. J. Song , and D. Adhia . 2022. “Tinnitus and the Triple Network Model: A Perspective.” Clinical and Experimental Otorhinolaryngology 15, no. 3: 205–212. 35835548 10.21053/ceo.2022.00815 PMC9441510

[brb371094-bib-0015] Deniz‐Sakarya, M. , M. Çınar‐Satekin , Z. Ç. B. Yaldız , and S. Tokgoz‐Yılmaz . 2024. “Does Chronic Subjective Tinnitus Affect Cognitive Performance in Adults With Hearing Thresholds of 25 dB and Less Between 0.5–4 kHz.” Journal of the American Academy of Audiology 35, no. 1/2: 40–46. 37989231 10.1055/a-2214-7927

[brb371094-bib-0016] Edwards, H. M. , J. G. Jackson , and H. Evans . 2022. “Neuroticism as a Covariate of Cognitive Task Performance in Individuals With Tinnitus.” Frontiers in Psychology 13: 865790. 10.3389/fpsyg.2022.906476 PMC9379139 35983209

[brb371094-bib-0017] Emadi, M. , A. Rasa, A. Moossavi , and M. Akbari . 2023a. “Combined Bifrontal Transcranial Direct Current Stimulation and Auditory Stroop Training in Chronic Tinnitus.” Indian Journal of Otolaryngology and Head & Neck Surgery 75, no. 1: 8–13. 37007882 10.1007/s12070-022-03258-z PMC10050537

[brb371094-bib-0018] Emadi, M. , A. Moossavi , M. Akbari , et al. 2023b. “Effect of Cognitive Rehabilitation by Auditory Stroop Training on Quality of Life of Individuals With Tinnitus.” Indian Journal of Otolaryngology and Head and Neck Surgery 75, no. 4: 3487–3492. 37974734 10.1007/s12070-023-04011-w PMC10645756

[brb371094-bib-0019] Emadi, M. , A. Moossavi , M. Akbari , S. Jalaie , and R. Toufan . 2022. “Testing a Hypothesis: Tinnitus Control by Enhancing Physiological Inhibition.” Indian Journal of Otolaryngology and Head and Neck Surgery 74, no. Suppl 3: 4212–4217. 36742728 10.1007/s12070-021-02915-z PMC9895585

[brb371094-bib-0020] Fetoni, A. R. , T. Di Cesare , S. Settimi , et al. 2021. “The Evaluation of Global Cognitive and Emotional Status of Older Patients With Chronic Tinnitus.” Brain and Behavior 11, no. 8: e02074. 34288570 10.1002/brb3.2074 PMC8413806

[brb371094-bib-0021] Ghodratitoostani, I. , O. A. Gonzatto Jr. , Z. Vaziri , et al. 2022. “Dose‐Response Transcranial Electrical Stimulation Study Design: A Well‐Controlled Adaptive Seamless Bayesian Method to Illuminate Negative Valence Role in Tinnitus Perception.” Frontiers in Human Neuroscience 16: 811550. 35677206 10.3389/fnhum.2022.811550 PMC9169505

[brb371094-bib-0022] Ghodratitoostani, I. , Y. Zana , A. C. B. Delbem , S. S. Sani , H. Ekhtiari , and T. G. Sanchez . 2016. “Theoretical Tinnitus Framework: A Neurofunctional Model.” Frontiers in Neuroscience 10: 114. 27594822 10.3389/fnins.2016.00370 PMC4990547

[brb371094-bib-0023] Golm, D. , C. Schmidt‐Samoa , P. Dechent , and B. Kröner‐Herwig . 2016. “Tinnitus‐related Distress: Evidence From fMRI of an Emotional Stroop Task.” BMC Ear, Nose and Throat Disorders 16, no. 1: 10. 27499700 10.1186/s12901-016-0029-1 PMC4975911

[brb371094-bib-0024] Gonendik, Z. A. , B. Mujdeci , S. E. Karakurt , and H. H. Dere . 2021. “Assessment of Stroop Color Word Interference Test–TBAG Form Performance in Subjects With Tinnitus.” European Archives of Oto‐Rhino‐Laryngology 278, no. 5: 1403–1409. 32710180 10.1007/s00405-020-06221-2

[brb371094-bib-0025] Grenier, B. , C. Berr , M. Goldberg , et al. 2024. “Hearing Loss, Hearing Aids, and Cognition.” JAMA Network Open 7, no. 10: e2436723. 39352700 10.1001/jamanetworkopen.2024.36723 PMC11445684

[brb371094-bib-0026] Haupt, S. , N. Axmacher , M. X. Cohen , C. E. Elger , and J. Fell . 2009. “Activation of the Caudal Anterior Cingulate Cortex due to Task‐related Interference in an Auditory Stroop Paradigm.” Human Brain Mapping 30, no. 9: 3043–3056. 19180558 10.1002/hbm.20731 PMC6870781

[brb371094-bib-0027] Heinrich, A. , and S. Knight . 2020. “Reproducibility in Cognitive Hearing Research: Theoretical Considerations and Their Practical Application in Multi‐lab Studies.” Frontiers in Psychology 11: 1590. 32765364 10.3389/fpsyg.2020.01590 PMC7378399

[brb371094-bib-0028] Inguscio, B. M. S. , D. Rossi , G. Giliberto , et al. 2024. “Bridging the Gap Between Psychophysiological and Audiological Factors in the Assessment of Tinnitus: An EEG Investigation in the Beta Band.” Brain Sciences 14, no. 6: 570. 38928570 10.3390/brainsci14060570 PMC11202302

[brb371094-bib-0029] Isler, B. , P. Neff , and T. Kleinjung . 2023. “Möglichkeiten der funktionellen bildgebung bei tinnitus [Functional neuroimaging options for tinnitus].” HNO 71, no. 10: 640–647. 37382658 10.1007/s00106-023-01319-5 PMC10520110

[brb371094-bib-0030] Jackson, J. G. , I. J. Coyne , and P. J. Clough . 2014. “A Preliminary Investigation of Potential Cognitive Performance Decrements in Non‐help‐seeking Tinnitus Sufferers.” International Journal of Audiology 53, no. 2: 88–93. 24191964 10.3109/14992027.2013.846481

[brb371094-bib-0031] James, G. A. , J. D. Thostenson , G. Brown , et al. 2017. “Neural Activity During Attentional Conflict Predicts Reduction in Tinnitus Perception Following rTMS.” Brain Stimulation 10, no. 5: 934–943. 28629874 10.1016/j.brs.2017.05.009 PMC5600281

[brb371094-bib-0032] Jarach, C. M. , A. Lugo , M. Scala , et al. 2022. “Global Prevalence and Incidence of Tinnitus: A Systematic Review and Meta‐Analysis.” JAMA Neurology 79, no. 9: 888–900. 10.1001/jamaneurol.2022.2189. 35939312 PMC9361184

[brb371094-bib-0033] Knight, S. , and A. Heinrich . 2017. “Different Measures of Auditory and Visual Stroop Interference and Their Relationship to Speech Intelligibility in Noise.” Frontiers in Psychology 8: 430. 28367129 10.3389/fpsyg.2017.00230 PMC5355492

[brb371094-bib-0034] Leong, S. L. , S. Tchen , I. H. Robertson , O. Alsalman , W. T. To , and S. Vanneste . 2020. “The Potential Interruptive Effect of Tinnitus‐related Distress on Attention.” Scientific Reports 10, no. 1: 11911. 32681139 10.1038/s41598-020-68664-1 PMC7367824

[brb371094-bib-0035] Logan, G. D. 1980. “Attention and Automaticity in Stroop and Priming Tasks: Theory and Data.” Cognitive Psychology 12, no. 4: 523–553. 7418368 10.1016/0010-0285(80)90019-5

[brb371094-bib-0036] MacLeod, C. M. 1991. “Half a Century of Research on the Stroop Effect: An Integrative Review.” Psychological Bulletin 109, no. 2: 163–203. 2034749 10.1037/0033-2909.109.2.163

[brb371094-bib-0037] Martins, M. E. O. , C. M. G. Tosi , B. P. Luz , L. H. Toresan , C. F. Carvalho , and N. M. Dias . 2023. “O Paradigma de Stroop Nos Estudos Brasileiros: Uma Revisão de Escopo.” Psicologia: Teoria e Prática 25, no. 2: e252174.

[brb371094-bib-0038] Mazurek, B. , G. Hesse , C. Dobel , V. Kratzsch , C. Lahmann , and H. Sattel , Guideline Group . 2022. “Chronic Tinnitus.” Dtsch Arztebl International 119, no. 13: 219–225. 10.3238/arztebl.m2022.0135 PMC9342131 35197187

[brb371094-bib-0039] McGowan, J. , M. Sampson , D. M. Salzwedel , E. Cogo , V. Foerster , and C. Lefebvre . 2016. “PRESS Peer Review of Electronic Search Strategies: 2015 Guideline Statement.” Journal of Clinical Epidemiology 75: 40–46. 27005575 10.1016/j.jclinepi.2016.01.021

[brb371094-bib-0040] Morse, K. , and L. Morse . 2025. “Convergent Validity of Cortical Auditory Evoked Potential Indices of central Auditory Nervous System Inhibition in People With and Without Tinnitus.” Hearing Research 458: 109185. 39893715 10.1016/j.heares.2025.109185

[brb371094-bib-0041] Noreña, A. J. , S. Lacher‐Fougère , M.‐J. Fraysse , et al. 2021. “A Contribution to the Debate on Tinnitus Definition.” Progress in Brain Research 262: 469–485. 33931192 10.1016/bs.pbr.2021.01.029

[brb371094-bib-0042] Sharma, A. , M. Mohanty , N. Panda , and S. Munjal . 2022. “Impact of Hearing Loss on Cognitive Abilities in Subjects With Tinnitus.” Neurology India 70, no. 2: 554–562. 35532619 10.4103/0028-3886.344654

[brb371094-bib-0043] Sharma, A. , M. Mohanty , N. Panda , and S. Munjal . 2023. “Neuropsychological Differences Between the Unilateral and Bilateral Tinnitus Participants With Normal Hearing.” Folia Phoniatrica Et Logopaedica 75, no. 2: 67–80. 35917799 10.1159/000526138

[brb371094-bib-0044] Sommerhalder, N. , P. Neff , Z. Bureš , O. Profant , T. Kleinjung , and M. Meyer . 2023. “Deficient Central Mechanisms in Tinnitus: Exploring the Impact on Speech Comprehension and Executive Functions.” Hearing Research 440: 108914. 37979435 10.1016/j.heares.2023.108914

[brb371094-bib-0045] Stevens, C. , G. Walker , M. Boyer , and M. Gallagher . 2007. “Severe Tinnitus and Its Effect on Selective and Divided Attention.” International Journal of Audiology 46, no. 5: 208–216. 17487668 10.1080/14992020601102329

[brb371094-bib-0046] Stroop, J. R. 1935. “Studies of Interference in Serial Verbal Reactions.” Journal of Experimental Psychology 18, no. 6: 643–662.

[brb371094-bib-0047] Tegg‐Quinn, S. , R. J. Bennett , R. H. Eikelboom , and D. M. Baguley . 2016. “The Impact of Tinnitus Upon Cognition in Adults: A Systematic Review.” International Journal of Audiology 55, no. 10: 533–540. 27240696 10.1080/14992027.2016.1185168

[brb371094-bib-0048] To, W. T. , D. De Ridder , T. Menovsky , J. Hart , and S. Vanneste . 2017. “The Role of the Dorsal Anterior Cingulate Cortex (dACC) in a Cognitive and Emotional Counting Stroop Task: Two Cases.” Restorative Neurology and Neuroscience 35, no. 3: 333–345. 28598859 10.3233/RNN-170730

[brb371094-bib-0049] Tricco, A. C. , E. Lillie , W. Zarin , et al. 2018. “PRISMA Extension for Scoping Reviews (PRISMA‐ScR): Checklist and Explanation.” Annals of Internal Medicine 169, no. 7: 467–473. 30178033 10.7326/M18-0850

[brb371094-bib-0050] Van Wilderode, M. , E. Vermaete , T. Francart , J. Wouters , and A. van Wieringen . 2023. “Effectiveness of Auditory Training in Experienced Hearing‐aid Users, and an Exploration of Their Health‐related Quality of Life and Coping Strategies.” Trends in Hearing 27: 23312165231198380. 10.1177/23312165231198380. 37709273 PMC10503297

[brb371094-bib-0051] Waechter, S. , and K. J. Brännström . 2015. “The Impact of Tinnitus on Cognitive Performance in Normal‐hearing Individuals.” International Journal of Audiology 54, no. 11: 845–851. 26107427 10.3109/14992027.2015.1055836

[brb371094-bib-0052] Waechter, S. , W. J. Wilson , and J. K. Brännström . 2021. “The Impact of Tinnitus on Working Memory Capacity.” International Journal of Audiology 60, no. 4: 274–281. 33000654 10.1080/14992027.2020.1822550

[brb371094-bib-0053] Waechter, S. , W. J. Wilson , M. Magnusson , and K. J. Brännström . 2022. “Extended High Frequency Hearing, but Not Tinnitus, Is Associated With Every‐Day Cognitive Performance.” Frontiers in Psychology 13: 913944. 35774957 10.3389/fpsyg.2022.913944 PMC9237571

[brb371094-bib-0054] Wang, Y. , J. Zhang , W. Hu , et al. 2018. “The Characteristics of Cognitive Impairment in Subjective Chronic Tinnitus.” Brain and Behavior 8, no. 3: e00918. 29541537 10.1002/brb3.918 PMC5840442

[brb371094-bib-0055] Washburn, D. A. 2016. “The Stroop Effect at 80: The Competition Between Stimulus Control and Cognitive Control.” Journal of the Experimental Analysis of Behavior 105, no. 1: 3–13. 26781048 10.1002/jeab.194

[brb371094-bib-0056] Webb, S. L. , V. Loh , A. Lampit , J. E. Bateman , and D. P. Birney . 2018. “Meta‐Analysis of the Effects of Computerized Cognitive Training on Executive Functions: A Cross‐Disciplinary Taxonomy for Classifying Outcome Cognitive Factors.” Neuropsychology Review 28, no. 2: 232–250. 29721646 10.1007/s11065-018-9374-8

[brb371094-bib-0057] Williams, V. , A. M. Boylan , and D. Nunan . 2020. “Critical Appraisal of Qualitative Research: Necessity, Partialities, and the Issue of Bias.” BMJ Evidence‐Based Medicine 25, no. 1: 9–11. 10.1136/bmjebm-2018-111132 30862711

